# Supplementary material for: SoxB1 family members inhibit Wnt signaling to promote maturation and deposition of stable neuromasts by the zebrafish Posterior Lateral Line primordium
Source: bioRxiv. 2025 Apr 26:2025.04.23.650055. Preprint. [Version 1] doi: 10.1101/2025.04.23.650055 (PMC12190404; doi:10.1101/2025.04.23.650055)
Supplement: 1 — Supplementary Figure 1. The relationship between sox1a and sox2 expression and Wnt activity, as indicated by lef1 A, B, D. sox1a expression (cyan) typically overlaps with lef1 expression (yellow). A, C, E. sox2 expression (magenta) is complementary to lef1 expression (yellow). F. sox1a (cyan) and sox2 (magenta) expressions are complementary to each other. In C, white arrow shows center-biased expression, blue arrow -broad expression, and black arrow shows donut shaped expression. Supplementary Figure 2. Cross-reactivity of sox2 MO1 with sox3 and enhanced phenotype compared to sox2-specific MO A. sox2 and sox3 antibodies labeling control, sox2 MO1 and sox3 morphant pLLP. B. L1 deposition in sox2 MO1 and sox2-specific morphants. Asterisks indicate significance by Mann-Whitney test (**** p< 0.0001, * p< 0.05). Supplementary Figure 3. L1 deposition in sox1a/sox2 and sox2/sox3 heterozygous mutant incross A, B. Deposition distance of L1 neuromast in 52hpf double mutant embryos. sox2+/− and sox1a+/− or sox3+/− were crossed to generate heterozygous progeny for imaging followed by genotyping. Asterisks indicate significance by Mann-Whitney test (*** p< 0.001, ** p< 0.01, * p< 0.05). Supplementary Figure 4. CRISPR mutagenesis of sox2y589 and sox1ay590 A. sox2 insertional mutant with site of frame shift and predicted translation. B. sox1a deletional mutant with site of frame shift and predicted translation. [file NIHPP2025.04.23.650055V1-supplement-1.pdf]

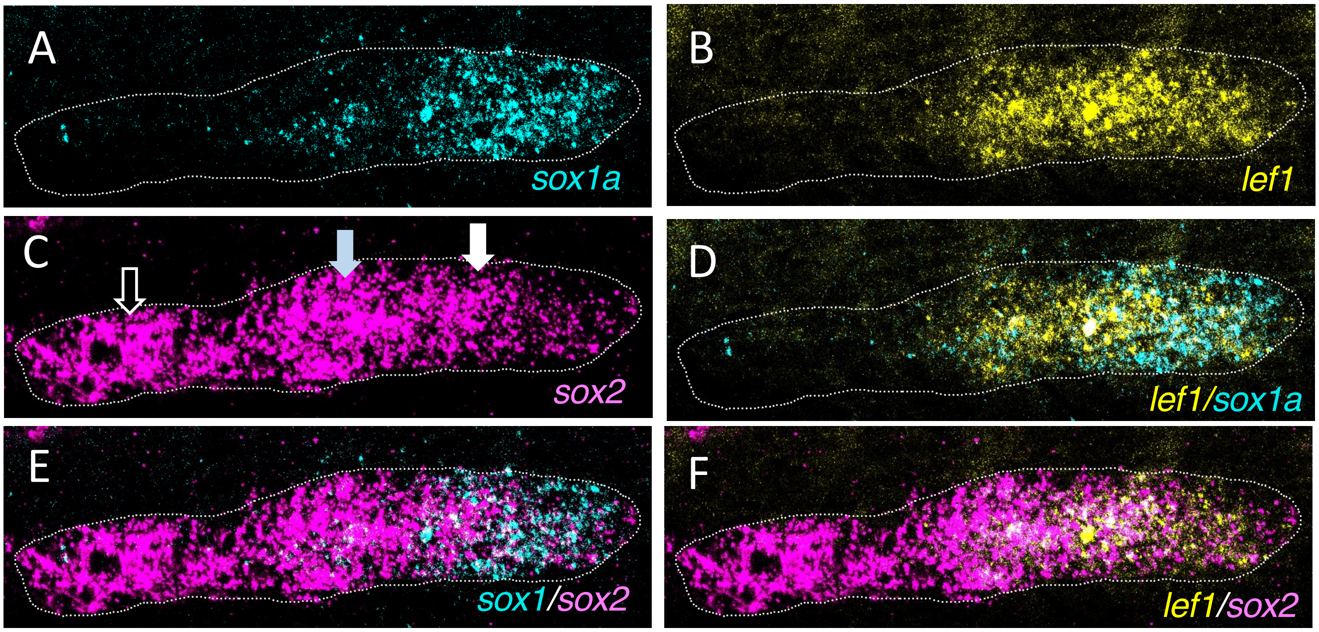

Relationship of *sox1a* and *sox2* expression to Wnt activity as defined by Lef1. sox1a expression (blue) typically overlaps with lef1 (yellow) , while it is complementary to sox2 (magenta).

Supplementary Figure 1

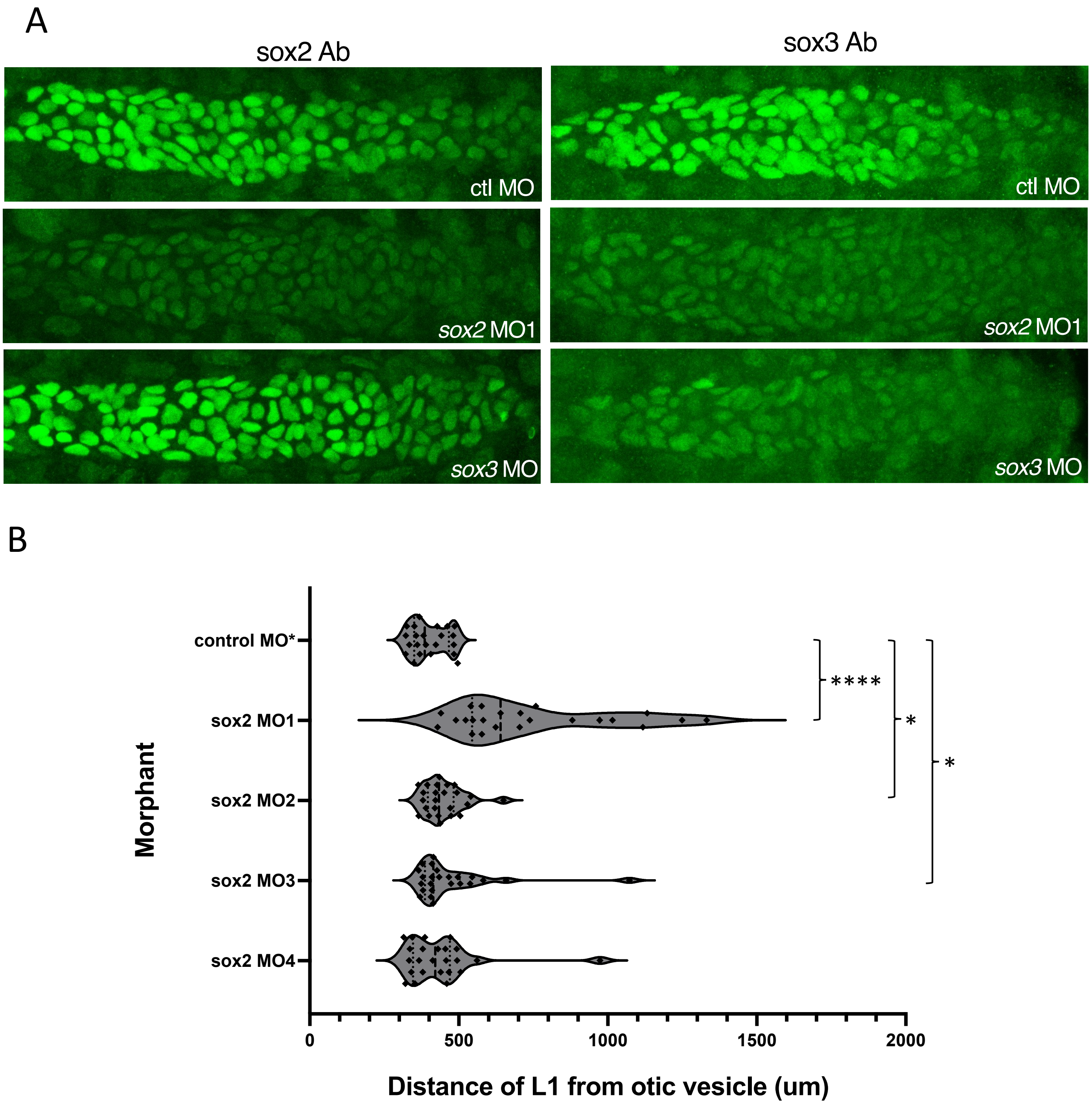

Cross-reactivity of sox2 MO1 with sox3 and enhanced phenotype compared to sox2-specific MO.

Supplemental Figure 2

A

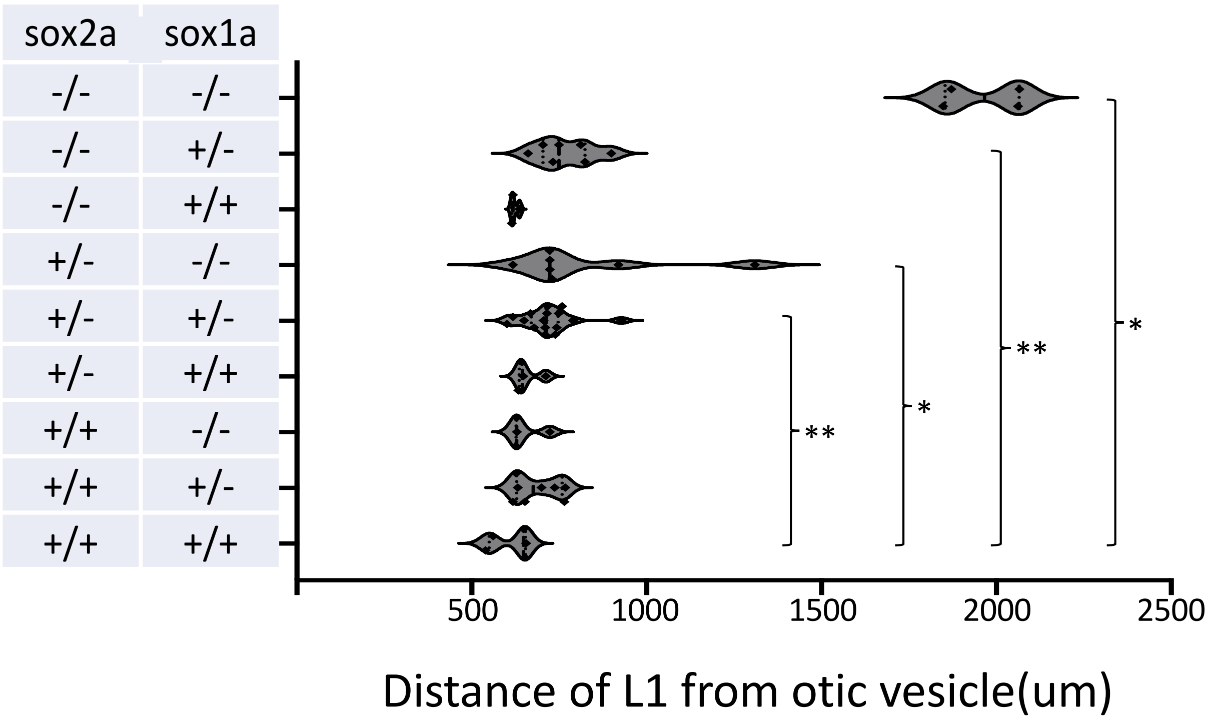

B

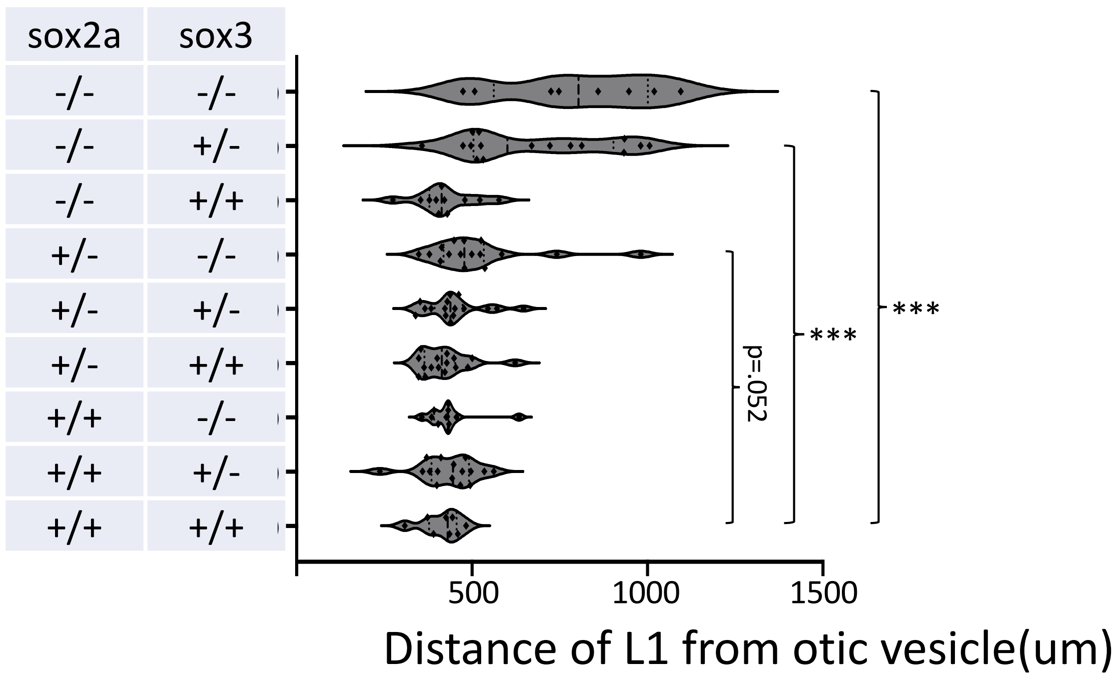

Supplementary Figure 3

A

CRISPR mutagenesis yields sox2 with 14bp insertion resulting in predicted frame shift and premature stop codon

sox2^{y589}

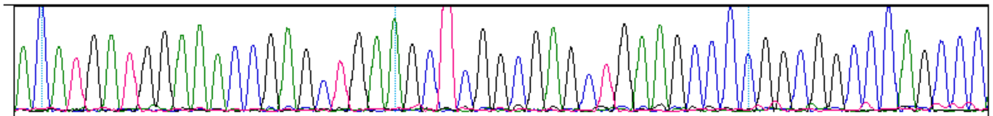

ACATGATGGAAACCGAGCTGAAGCTCGGCGAGCTGAAGCCCCGGCGGCCCAGCCC

sox2 acatgatggaaaccgagctgaagCCCC-----C-G--G---C-GC--CCCAGCCC

CRISPR target GGAAACCGAGCTGAAGCCCC

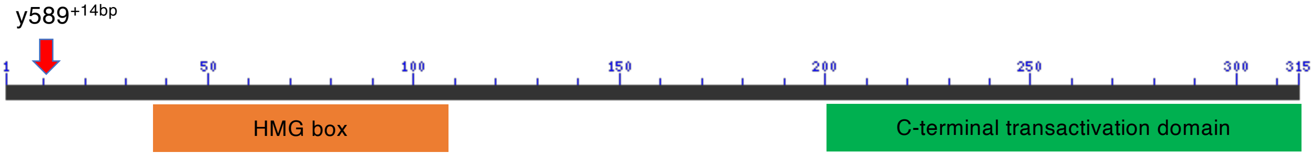

P11L

predicted sox2^{y589} MYNMMETELKLGELKPRRPSPTPGARGTPTPRETTRKTARTASRDP.

B

CRISPR mutagenesis yields sox1a with 13bp deletion resulting in predicted frame shift and premature stop codon

sox1a^{y590} TGGAAACGGACCTTCATTC-------------ACCAACACGAACCCGGGGCAAACGGGTC

sox1a TGGAAACGGACCTTCATTCCCCGGGACCCCAAACCAACACGAACCCGGGGCAAACGGGTC

CRISPR target GGAAACGGACCTTCATTCCCCGG

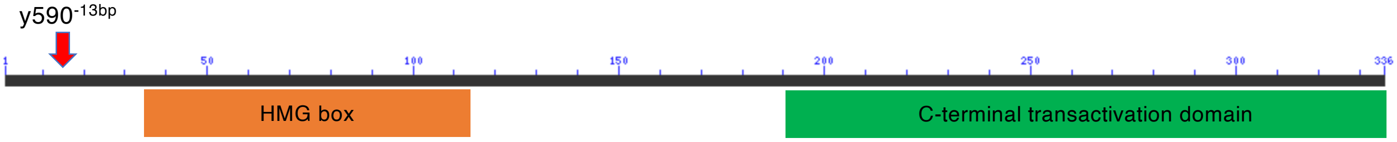

G11T

predicted sox1a^{y590} MYSMMMETDLHSPTRTRGKRVQIRGAKPIKTA.

Supplementary Figure 4
